# Dwarfing Genes *Rht-B1b* and *Rht-D1b* Are Associated with Both Type I FHB Susceptibility and Low Anther Extrusion in Two Bread Wheat Populations

**DOI:** 10.1371/journal.pone.0162499

**Published:** 2016-09-08

**Authors:** Xinyao He, Pawan K. Singh, Susanne Dreisigacker, Sukhwinder Singh, Morten Lillemo, Etienne Duveiller

**Affiliations:** 1 International Maize and Wheat Improvement Center (CIMMYT), Apdo. Postal 6–641, 06600 Mexico DF, Mexico; 2 Department of Plant Sciences, Norwegian University of Life Sciences, P.O. Box 5003, NO-1432 Ås, Norway; Institute of Genetics and Developmental Biology Chinese Academy of Sciences, CHINA

## Abstract

It has been well documented that dwarfing genes *Rht-B1b* and *Rht-D1b* are associated with Type I susceptibility to Fusarium head blight (FHB) in wheat; but the underlying mechanism has not been well delineated. Anther extrusion (AE) has also been related to Type I resistance for initial FHB infection, where high AE renders FHB resistance. In this study, two doubled haploid populations were used to investigate the impact of the two dwarfing genes on FHB resistance and AE, and to elucidate the role of AE in *Rht-*mediated FHB susceptibility. Both populations were derived by crossing the FHB susceptible cultivar ‘Ocoroni F86’ (*Rht-B1a*/*Rht-D1b*) with an FHB resistant variety (*Rht-B1b*/*Rht-D1a*), which was ‘TRAP#1/BOW//Taigu derivative’ in one population (the TO population) and ‘Ivan/Soru#2’ in the other (the IO population). Field experiments were carried out from 2010 to 2012 in El Batán, Mexico, where spray inoculation was adopted and FHB index, plant height (PH), and AE were evaluated, with the latter two traits showing always significantly negative correlations with FHB severity. The populations were genotyped with the DArTseq GBS platform, the two dwarfing genes and a few SSRs for QTL analysis, and the results indicated that *Rht-B1b* and *Rht-D1b* collectively accounted for 0–41% of FHB susceptibility and 13–23% of reduced AE. It was also observed that three out of the four AE QTL in the TO population and four out of the five AE QTL in the IO population were associated with FHB resistance. Collectively, our results demonstrated the effects of *Rht-B1b* and *Rht-D1b* on Type I FHB susceptibility and reducing AE, and proposed that their impacts on Type I FHB susceptibility may partly be explained by their effects on reducing AE. The implication of the relationship between the two dwarfing genes and AE for hybrid wheat breeding was also discussed.

## Introduction

Fusarium head blight (FHB) is a notorious wheat disease prevailing in warm and humid environments, exerting global impact on food and feed safety due to the presence of mycotoxins produced by *Fusarium* species, the causal agents of FHB [1, 2]. Deoxynivalenol (DON) has been considered the most important FHB-related mycotoxin and legislation has been set up in many countries/organizations for controlling DON content in food and feed [3].

Host resistance to FHB is of quantitative inheritance and influenced significantly by environment [4], making breeding for this trait a difficult task. Multiple mechanisms of host resistance to FHB has been recognized, including Type I for resistance to initial infection, Type II for spread of pathogen in spike tissues, Type III for DON accumulation, Type IV for kernel infection, and Type V for yield reduction [5, 6]. In relation to food safety, Type III resistance is the most important; but so far no validated QTL specific for this resistance mechanism has been identified [7], and some researchers still regard it as a consequence of FHB infection and not an independent trait [1]. Of the first two resistance mechanisms, Type I resistance exhibited more frequent association with phenological, morphological, and flower biology traits, such as days to heading (DH), plant height (PH) and anther extrusion (AE) [8–11].

The negative association between PH and FHB susceptibility in wheat has long been observed, and it happened also in barley and oat [12–14]. Three possible mechanisms have been proposed for the association, i.e. disease escape, pleiotropy of reduced height (*Rht*) genes, and tight linkage [3]. In the last decade researches provided molecular evidence for this relationship and several QTL responsible for both FHB and PH were identified, including *Rht-B1*, *Rht-D1* and *Rht8* [9]. Dwarfing genes *Rht-B1b* and *Rht-D1b* (formally known as *Rht1* and *Rht2*, respectively) were derived from the Japanese cultivar ‘Norin 10’ and contributed greatly to the Green Revolution [15]. Strong evidences are available for the association between *Rht-D1b* and Type I FHB susceptibility in European varieties [16–20]. For example, *Rht-D1b* increased FHB severity by 52% in a ‘Mercia’ background and 38% in a ‘Maris Huntsman’ background [20]. Lu et al. [21] demonstrated in a mapping population that two major resistant QTL may be required to counteract the negative effect of *Rht-D1b*. In an association mapping study on European winter wheat materials, Miedaner et al. [22] also reported the significant association of *Rht-D1b* with increased FHB susceptibility, but to a lesser degree than reported previously; the authors concluded that the negative effects of *Rht-D1b* in bi-parental populations may have been overestimated. In the case of *Rht-B1b*, Srinivasachary et al. [23] found that it showed little or no negative impact on Type I FHB resistance under moderate FHB pressure, but exerted negative effects similar to *Rht-D1b* under severe infection. Miedaner and Voss [20] also reported the different performance of *Rht-B1b* under different genetic backgrounds. In the three mapping populations tested by Buerstmayr et al. [24], *Rht-B1b* was associated with increased FHB susceptibility, with phenotypic effects ranging from 3–18%. The negative effects of the two dwarfing genes on field FHB resistance have also been reported in Chinese and US wheat materials [25, 26]. In point inoculated experiments, *Rht-B1b* exhibited significant effects on Type II resistance, whereas *Rht-D1b* showed little or no effects on this type of FHB resistance [19, 23, 26–28]. Many researchers ascribed the association to the pleiotropic effects of dwarfing genes [17, 19, 24]; but Yan et al. [28] claimed that it was the micro-environmental condition around spikes that contributed to the relationship, since the negative effects on Type I resistance disappeared when *Rht-B1b* and *Rht-D1b* near-isogenic lines were physically raised to the same heights as their tall counterparts.

The importance of anthers in FHB infection has long been observed. Pugh et al. [29] observed that retained anthers were the first tissues to be colonized as a base for further infection. Strange and Smith [30] also found this phenomenon and reported that the presence of anthers favoured greatly the FHB infection, whereas emasculation significantly reduced the disease severity. They ascribed this to the fungal growth stimulants in anthers, of which choline and betaine were the two major components [31]. Based on these findings, Strange et al. [32] suggested the selection of wheat lines with low anther retention (or high AE) to facilitate FHB resistance breeding. Three decades later, Skinnes et al. [33] and Graham and Browne [34] reported the association of FHB with AE in European wheat varieties, where those having high AE tended to had low FHB severity. The positive correlation between AE and FHB resistance has also been reported in Chinese, Japanese and CIMMYT germplasm [35–37]. QTL mapping studies revealed the underlying mechanisms for this relationship by identifying linked or coincided QTL for the two traits [8, 11, 38, 39]. Like PH, AE was also found to be associated with Type I FHB resistance [11].

Considering the associations of Type I FHB resistance with both PH and AE, it is tempting to investigate the association between the latter two traits, and clues do exist in literature. In the Shanghai-3/Catbird x Naxos population, *Rht-B1* explained 10% of the phenotypic variation of AE [11], and in the Hermann × Skalmeje population, lines with *Rht-B1b* or *Rht-D1b* showed reduced AE and double dwarfs (*Rht-B1b*/*Rht-D1b*) had a high degree of anther retention (+99%) compared to tall lines with *Rht-B1a*/*Rht-D1a* [40]. It was also observed by hybrid wheat breeders that PH and AE are positively correlated [41]. The objectives of the current study were to map QTL for FHB and its related traits and to evaluate the impacts of dwarfing genes *Rht-B1b* and *Rht-D1b* on field FHB resistance and AE in two mapping populations.

## Materials and Methods

### Plant material

Two doubled haploid populations were used in this study. The first one was developed from a cross between ‘TRAP#1/BOW//Taigu derivative’ and ‘Ocoroni F86’ with 135 progenies (referred to as the TO population hereafter), while the second was from ‘Ivan/Soru#2’ and ‘Ocoroni F86’ with 92 progenies (the IO population). Both the two female parents were FHB resistant lines bred at CIMMYT, while ‘Ocoroni F86’ (pedigree JUPATECO-73/(SIB)EMU//(SIB)GRAJO) is a CIMMYT breeding line moderately susceptible to FHB [42]. Both of the two resistant parents carried *Rht-B1b*/*Rht-D1a* whereas the susceptible parent had the *Rht-B1a*/*Rht-D1b* genotype, resulting in both dwarfing genes segregating in the two populations.

### Field trials and phenotyping

The field FHB experiments were conducted at the El Batán experimental station (altitude of 2,240 meters above sea level, coordinate 19.5°N, 98.8°W, with an average annual precipitation of 625 mm) of CIMMYT, Mexico, during the summer season (May to September) when rainfall is concentrated. The two populations were evaluated from 2010 to 2012, sown in 1 m double rows with randomized complete block design with three replications. Each year, a mixture of 5 aggressive *F*. *graminearum* isolates were collected, characterized, and used for field inoculation, following the protocols described by He et al. [42]. Spray inoculation was targeted to each line’s anthesis stage with an inoculum of 50,000 spores/ml and was repeated two days later. From anthesis to early dough stages, the nursery was misted from 9am to 8pm with 10 minutes of spraying each hour, to create a humid environment favourable for FHB development. A wheat/maize rotation and conservation agricultural practices were followed in the nursery to enhance natural inoculum.

FHB symptoms were evaluated at 25 days post inoculation (dpi) on the 10 spikes that had been tagged at anthesis. Numbers of infected spikes and symptomatic spikelets of each spike were counted for calculating FHB index with the formula: *FHB index* = *Severity* x *Incidence* [43], where *Severity* stands for the averaged percentage of diseased spikelets, and *Incidence* for the percentage of symptomatic spikes. Plots were sickle harvested and threshed with a belt thresher set at low wind speed to retain scabby kernels. Fusarium damaged kernels (FDK) was estimated only in 2012 for the two populations through visually evaluating a random sample in a petri dish, where both scabby and shrivelled kernels were regarded as FDK. DON content was quantified in 2010 and 2012 for the TO population and in 2011 and 2012 for the IO population, based on 2 g flour sampled from 20 g ground grain of each accession, using the Ridascreen Fast DON ELISA kit (RBiopharm GmbH, Darmstadt, Germany) following the manufacturer’s instructions. AE and PH were scored in 2011 and 2012 for the IO population and in 2012 for the TO population. In 2015, the TO population was planted in 40x15 cm hill plots with two replications for an additional evaluation of AE and PH. AE was rated with a linear scale from 0 (no extrusion) to 9 (full extrusion) according to Skinnes et al. [8], and PH was measured before harvest from ground to the average spike tips excluding awns in each plot. Days to heading (DH) was scored for the two populations in all the experiments.

### Statistical analyses

The phenotypic data was analysed by the SAS program ver. 9.2. Analysis of variance (ANOVA) was carried out with the PROC GLM module, and Pearson correlation coefficients were calculated using the PROC CORR function. The results of ANOVA were used for calculating the heritability estimates, using the formula $h^{2}=\sigma_{g}^{2}/(\sigma_{g}^{2}+\frac{\sigma_{e}^{2}}{r})$ for single years and $h^{2}=\sigma_{g}^{2}/(\sigma_{g}^{2}+\frac{\sigma_{g*y}^{2}}{y}+\frac{\sigma_{e}^{2}}{ry})$ for multiple years; in which $\sigma_{g}^{2}$ stands for genetic variance, $\sigma_{g*y}^{2}$ for genotype-by-year interaction, $\sigma_{e}^{2}$ for error variance, *y* for the number of years, and *r* for the number of replications [11].

### Genotyping

The two populations were genotyped with the DArTseq genotyping-by-sequencing (GBS) platform at the Genetic Analysis Service for Agriculture (SAGA) in Guadalajara, Mexico. This genotyping method is a combination of complexity reduction methods developed for array-based DArT and sequencing of resulting representations on next-generation sequencing platforms, for detailed information please check Li et al. [44]. Additionally, two dwarfing genes *Rht-B1* and *Rht-D1* were also genotyped, using the KASPar technology (KBioscience) based SNP markers developed at CIMMYT [45]. A few SSR markers linked to known FHB resistance QTL [7] were also applied. Markers with missing data points greater than 20% and segregation ratio beyond the range 0.5–2.0 were discarded from further analysis.

### Linkage and QTL analysis

Linkage groups (LGs) were constructed using the JoinMap v.4 software [46], where groupings were based on LOD values from 5 to 10, and ordering within each LG was done with the Maximum Likelihood algorithm. LGs were assigned to chromosomes according to the consensus GBS map by Li et al. [44]. QTL mapping was carried out with MapQTL v6.0 [47], in which interval mapping (IM) was first performed to detect potential QTL for each trait, followed by multiple QTL mapping (MQM) for each QTL, using the closest linked markers to each QTL detected in IM as cofactors. QTL were taken as significant and were reported if they were over the LOD threshold of 3 in at least one environment or over the threshold of 2 in multiple environments. LGs and LOD curves were drawn by the software MapChart ver. 2.3 [48].

## Results

**Fig 1:**
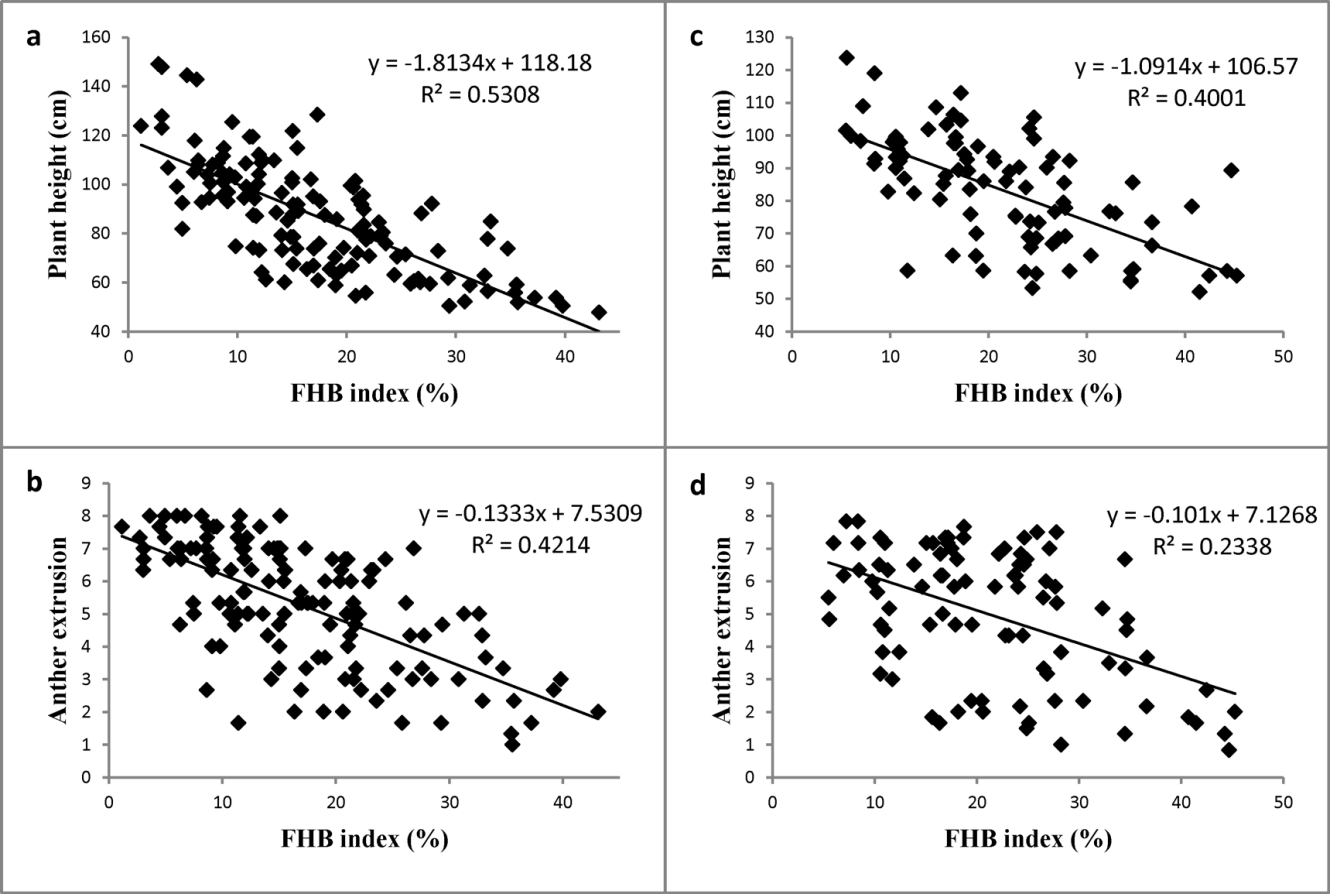
**Scatter plots of FHB index against plant height and anther extrusion in the ‘TRAP#1/BOW//Taigu derivative’ x ‘Ocoroni F86’ (a-b) and ‘Ivan/Soru#2’ x ‘Ocoroni F86’ (c-d) populations based on overall means.** The correlations are all significant at *p*<0.0001.

FHB development of the two populations was satisfactory, ranging from slight infection to around 50% of FHB index in all the three years (Fig 1). The resistant parents ‘TRAP#1/BOW//Taigu derivative’ and ‘Ivan/Soru#2’ showed always significantly higher resistance than the susceptible parent ‘Ocoroni F86’, in terms of all three FHB parameters. In both populations, ‘year’ effect contributed the most variation of FHB and DON, followed by ‘genotype’ and ‘genotype x year’ effects which were also significant except for DON in the TO population (Table 1). Usually high heritability estimates were obtained for the FHB parameters, but DON in the TO population had a value of merely 0.22 (Table 1). Significantly positive correlations were found among all the FHB traits, although in several cases the *r* values were low (Table 2).

**Table 1:** Analysis of variance for Fusarium head blight and associated traits and their heritability estimates in the ‘TRAP#1/BOW//Taigu derivative’ x ‘Ocoroni F86’ (TO) and ‘Ivan/Soru#2’ x ‘Ocoroni F86’ (IO) populations.

	Traits	Source	DF	Mean square	*F* value	*P* value	Heritability
**TO**	FHB	Genotype	138	743.80	6.27	<0.0001	0.84
		Year	2	15025.92	126.62	<0.0001	
		Genotype x Year	275	118.67	3.07	<0.0001	
		Rep (Year)	6	280.06	7.24	<0.0001	
		Error	811	38.70			
	DON	Genotype	138	41.83	1.26	0.0879	0.22
		Year	1	1583.00	47.71	<0.0001	
		Genotype x Year	138	33.18	1.35	0.0121	
		Rep (Year)	3	210.54	8.59	<0.0001	
		Error	413	24.51			
	FDK	Genotype	129	1182.55	9.16	<0.0001	0.89
		Rep	1	35.53	0.28	0.6008	
		Error	126	129.13			
	Anther extrusion	Genotype	138	16.19	4.67	<0.0001	0.79
		Year	1	29.38	8.47	<0.0001	
		Genotype x Year	138	3.47	3.47	<0.0001	
		Rep (Year)	3	0.24	0.24	0.8673	
		Error	414	1.00			
	Plant height	Genotype	138	2276.49	37.67	<0.0001	0.97
		Year	1	675.22	11.17	<0.0001	
		Genotype x Year	138	60.44	4.26	<0.0001	
		Rep (Year)	3	161.13	11.37	<0.0001	
		Error	414	14.18			
**IO**	FHB	Genotype	93	602.42	3.90	<0.0001	0.74
		Year	2	6630.69	42.90	<0.0001	
		Genotype x Year	186	154.57	3.73	<0.0001	
		Rep (Year)	6	112.33	2.71	0.0133	
		Error	546	41.42			
	DON	Genotype	93	8.06	2.48	<0.0001	0.62
		Year	1	65.38	20.11	<0.0001	
		Genotype x Year	93	3.25	2.48	<0.0001	
		Rep (Year)	3	25.59	19.52	<0.0001	
		Error	269	1.31			
	FDK	Genotype	93	2460.63	8.25	<0.0001	0.88
		Rep	2	1410.71	4.73	0.0100	
		Error	177	298.13			
	Anther extrusion	Genotype	93	25.11	6.04	<0.0001	0.87
		Year	1	25.11	6.24	<0.0001	
		Genotype x Year	93	4.16	4.30	<0.0001	
		Rep (Year)	4	1.75	1.81	0.1266	
		Error	372	0.97			
	Plant height	Genotype	93	1685.82	35.60	<0.0001	0.98
		Year	1	7634.09	161.23	<0.0001	
		Genotype x Year	93	47.35	2.81	<0.0001	
		Rep (Year)	4	185.38	11.01	<0.0001	
		Error	372	16.84			

**Table 2:** Pearson correlation coefficients among FHB traits in the ‘TRAP#1/BOW//Taigu derivative’ x ‘Ocoroni F86’ (TO, below the diagonal) and ‘Ivan/Soru#2’ x ‘Ocoroni F86’ (IO, above the diagonal) populations.

	FHB10	DON10(11)	FHB11	FHB12	DON12	FDK12
FHB10	1	0.35*	0.48**	0.37**	0.52**	0.36*
DON10(11)^a^	0.62**	1	0.67**	0.54**	0.42**	0.42**
FHB11	0.72**	0.49**	1	0.61**	0.35*	0.56**
FHB12	0.61**	0.36**	0.65**	1	0.55**	0.74**
DON12	0.44**	0.35**	0.43**	0.61**	1	0.36*
FDK12	0.56**	0.29*	0.65**	0.69**	0.56**	1

* *P*<0.01

** *P*<0.0001

^a^ DON10 in the case of the TO population and DON11 in the case of the IO population.

AE and PH showed wide segregation in both populations (Fig 1). High heritability estimates of 0.79 and 0.87 were obtained for AE in the TO and IO populations, respectively, and in the case of PH the values were even higher (Table 1). The two traits showed significantly negative correlations with FHB in the TO population (*r* = -0.73 for PH vs. FHB, and *r* = -0.65 for AE vs. FHB, *p*<0.0001), and the corresponding correlations were also significant in the IO population but with lower *r* values (*r* = -0.63 for PH vs. FHB, and *r* = -0.48 for AE vs. FHB, *p*<0.0001).

In the TO population, 1,858 GBSs together with seven SSRs and the two dwarfing genes were used for LG construction. Thirty three LGs were generated, covering 4,053cM with an average density of 2.2 cM/marker. All but 1D chromosome were represented in this map and three LGs were not assigned to a chromosome due to a lack of anchored markers. Regarding the IO population, 1,986 GBSs, *Rht-B1*, *Rht-D1* and four SSRs were used for linkage mapping and 35 LGs were obtained. Total length of the LGs was 4,430cM with a very similar density as that of the TO population. In this case, only chromosome 6D was not represented and six LGs were not assigned to chromosomes.

**Fig 2:**
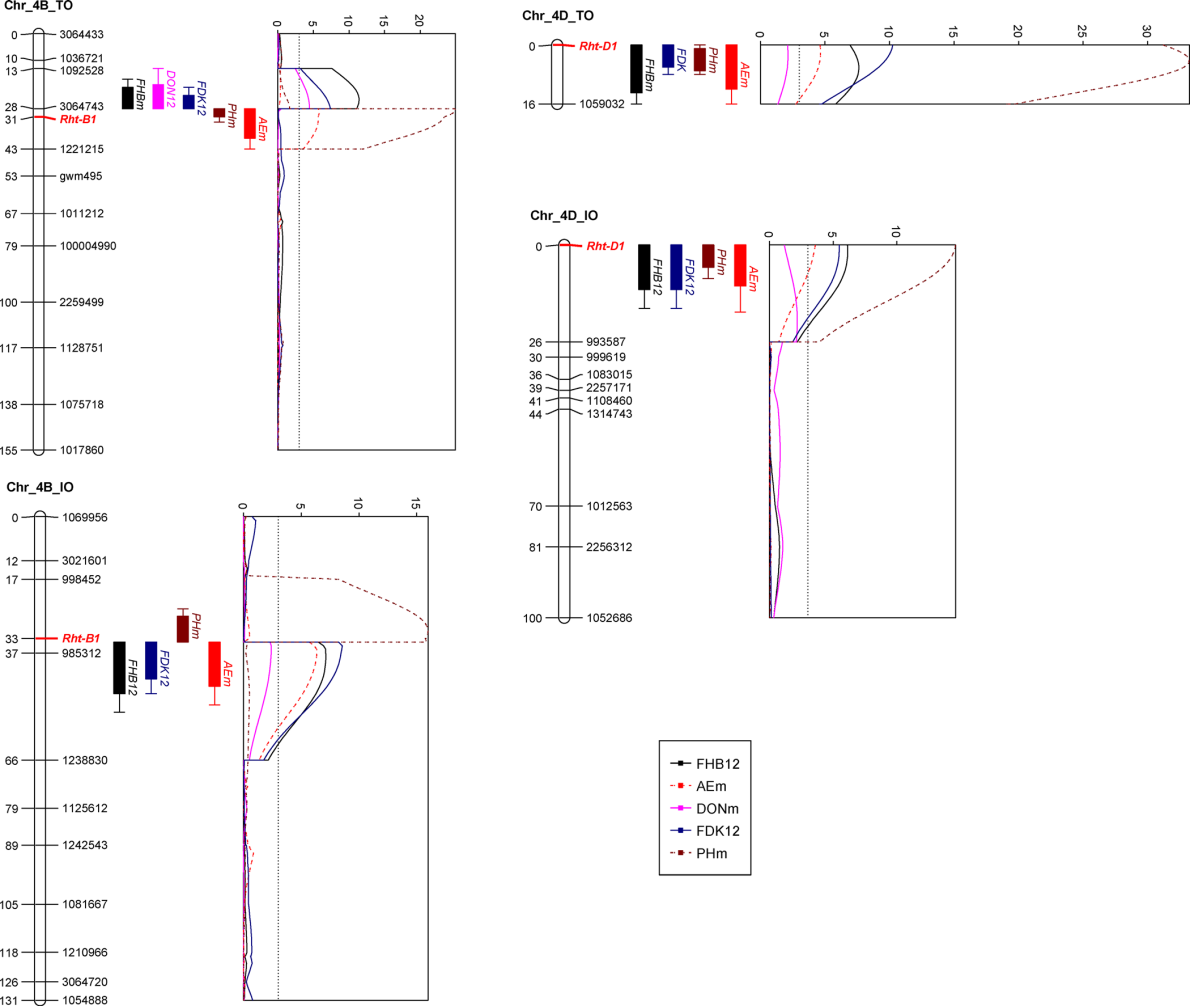
QTL profiles for FHB parameters, plant height and anther extrusion at the loci *Rht-B1* and *Rht-D1* based on mean phenotypic data in the ‘TRAP#1/BOW//Taigu derivative’ x ‘Ocoroni F86’ (TO) and ‘Ivan/Soru#2’ x ‘Ocoroni F86’ (IO) populations. If there was no QTL detected based on the mean, the environment with significant QTL effect was marked instead, with the year behind the QTL name. Genetic distances are shown in centimorgans to the left of the chromosomes. A threshold of 3.0 is indicated by a dashed vertical line in the LOD graphs. Only framework markers are presented except for the QTL regions, and the two dwarfing genes are highlighted in red.

Three QTL with major effects were identified in both populations, i.e. *Rht-B1* on 4BS, *Rht-D1* on 4DS and a QTL on 5AL (Table 3, Fig 2). The latter was most likely at *Vrn-A1*, due to its strong effects on heading time, explaining 48.2% of the DH variation in the TO population and 14.9% in the IO population. The expression of the three QTL was more stable in the TO population, being associated with FHB parameters in most environments, accounting mostly 10–20% of phenotypic variation. Comparably, the magnitude of their phenotypic effects was similar in the IO population, but their expression was not detected in certain environments, e.g. the 5AL QTL was not identified in 2012 and the *Rht-B1* QTL was significant mainly in 2012. The two dwarfing genes showed similar negative effects on FHB resistance in the two populations, although the phenotypic variations explained by *Rht-B1* were often a bit higher than those by *Rht-D1* (Table 3). A QTL on 2AL was also shared by the two populations based on common markers, but it was only a minor QTL accounting for phenotypic variations less than 10% (Table 3). Additional minor QTL were found in the IO population, located on 1BL, 3BL (LG 3B_2), 3BS (LG 3B_3) and 5BL (Table 3). It could be observed that for DON in the TO population, QTL on 2AL and 5AL in 2010 were significant, but the ones at *Rht-B1* and *Rht-D1* were identified in 2012 (Table 3), explaining the non-significant ‘genotype’ effect and low heritability estimate for this trait (Table 1).

**Table 3:** QTL for FHB traits after spray inoculations in the ‘TRAP#1/BOW//Taigu derivative’ x ‘Ocoroni F86’ (TO) and ‘Ivan/Soru#2’ x ‘Ocoroni F86’ (IO) populations and their association with other traits.

	Linkage group	Position	Left marker	Right marker	FHB index				FDK	DON content			R source^b^	Traits associated^c^
					2010	2011	2012	Mean	2012	2010^a^	2012	Mean		
**TO**	2A	78.4–90.4	1219210	1004513	2.6	3.4	3.3	3.8	2.7	4.7	** **		T	AE
	4B	13.4–30.5	1092528	Rht-B1	**11.2**	**13.7**	**15.2**	**16.6**	**14**		**12.9**		O	PH, AE
	4D	0–16.5	Rht-D1	1059032	** **	**7.5**	**15.7**	**9.6**	**20.4**	** **	5.8		T	PH, AE
	5A	62.0–72.4	1129347	2260918	**29.6**	**17.5**	**7.0**	**21.2**	5.4	**17.3**			T	DH, PH
	Accumulated percentage of variation explained				43.4	42.1	41.2	51.2	42.5	22.0	18.7			
**IO**	1B	17.7–22.6	100007924	1024654	5.3	**8.2**	** **	**6.5**		** **	** **	** **	I	
	2A	124.1–131.3	1027267	1694741		** **	6.5	** **		**7.8**	7.6	7.3	I	AE
	3B_2	53.5–82.1	997675	2268570						2.5	**6.4**	5.7	I	AE
	3B_3	78.6–85.0	1001892	2278701		**9.2**		**7.4**			** **	** **	O	
	4B	33.2–65.5	Rht-B1	1238830	** **	** **	**20.4**	** **	**25.4**	7.9	** **	6.6	O	PH, AE
	4D	0.0–26.4	Rht-D1	993587	** **	3.7	**17.1**	5.9	**15.2**	4.8	3.7	5.7	I	PH, AE
	5A	238.8–275.1	1067537	3064895	**12.2**	**21.1**	** **	**17.1**		**13.7**		7.6	I	DH
	5B	156.7–168.6	1066241	1216740	3.7	3.7	2.3	5.4	5.4				I	
	Accumulated percentage of variation explained				21.2	45.9	46.3	42.3	46	36.7	17.7	35.9		

The percentage of explained phenotypic variation in the multiple regression models is shown, QTL are listed if they were over the LOD threshold of 3 (in bold) in at least one environment or over the threshold of 2 in multiple environments.

^**a**^ In the case of the IO population, DON content was measured in 2011

^**b**^ *T* ‘TRAP#1/BOW//Taigu derivative’, *I* ‘Ivan/Soru#2’, *O* ‘Ocoroni F86’

^**c.**^ *AE* anther extrusion, *PH* plant height, *DH* days to heading.

Several QTL for AE were localized and three were shared by the two populations, viz. *Rht-B1*, *Rht-D1* and a QTL on 2AL (Table 4, Fig 2), all associated with FHB resistance (Table 3). Additional QTL were found on 2BL in the TO population and on 2DS and 3BL in the IO population (Table 4). The two dwarfing genes collectively explained around 20% of AE reduction in both populations and *Rht-B1b* was always more strongly associated with reduced AE than *Rht-D1b* (Table 4). As for PH, *Rht-B1* and *Rht-D1* collectively accounted for about 60% variation in the two populations, while additional QTL were found on 5AL (*Vrn-A1*) and 7B in the TO population and on 5BS in the IO population (Table 5).

**Table 4:** QTL for anther extrusion in the ‘TRAP#1/BOW//Taigu derivative’ x ‘Ocoroni F86’ (TO) and ‘Ivan/Soru#2’ x ‘Ocoroni F86’ (IO) populations.

	Linkage group	Position	Left marker		Right marker	Anther extrusion^a^			Source of elongation^b^
						2012	2015	Mean	
**TO**	2A	100.6–102.1	984869		3064488	**9.8**	**7.8**	**10.5**	T
	2B	244.0–247.0	1127943		1125516	5.2	4.3	**5.6**	T
	4B	28.5–31.3	3064743		Rht-B1	**10.1**	**8.8**	**11.1**	O
	4D	0–16.5	Rht-D1		1059032	**8.4**	**6.6**	**8.7**	T
	Accumulated percentage of variation explained					33.5	27.5	35.9	
**IO**	2A	117.0–128.1	1128135		1019498	**11.8**	**6.9**	**10.6**	I
	2D	4.5–5.6	2261713		984698	3.8	5.4	5.4	O
	3B_2	53.5–82.1	997675		2268570	6.9	**10**	**9.7**	I
	4B	33.2–65.5	Rht-B1		1238830	**7.8**	**16.2**	**13.2**	O
	4D	0.0–26.4	Rht-D1		993587	5.2	**6.9**	**6.9**	I
	Accumulated percentage of variation explained					35.5	45.4	45.8	

The percentage of explained phenotypic variation in the multiple regression models is shown, QTL are listed if they were over the LOD threshold of 3 (in bold) in at least one environment or over the threshold of 2 in multiple environments.

^a^ In the case of the IO population, AE was evaluated in 2011 and 2012

^b^ *T* ‘TRAP#1/BOW//Taigu derivative’, *I* ‘Ivan/Soru#2’, *O* ‘Ocoroni F86’.

**Table 5:** QTL for plant height in the ‘TRAP#1/BOW//Taigu derivative’ x ‘Ocoroni F86’ (TO) and ‘Ivan/Soru#2’ x ‘Ocoroni F86’ (IO) populations.

	Linkage group	Position	Left marker	Right marker	Plant height^a^			Source of tallness^b^^ ^
					2012	2015	Mean	
**TO**	4B	28.5–31.3	3064743	Rht-B1	**20.5**	**25.6**	**22.7**	O
	4D	0–16.5	Rht-D1	1059032	**35.4**	**35.9**	**35.1**	T
	5A	70.9–71.7	1135154	2262549	**14.3**	**5.2**	**9.6**	T
	7B	87.3–97.8	1081730	977335	** **	**3.5**	**3.2**	O
	Accumulated percentage of variation explained				70.2	70.2	70.6	
**IO**	4B	16.8–33.2	998452	Rht-B1	**30.4**	**30.8**	**31.1**	O
	4D	0–26.4	Rht-D1	993587	**28.7**	**25.2**	**27.6**	I
	5B	0–1.68	2282143	1255587	**5.0**	3.0	**4.0**	I
	Accumulated percentage of variation explained				64.1	59	62.7	

The percentage of explained phenotypic variation in the multiple regression models is shown, QTL are listed if they were over the LOD threshold of 3 (in bold) in at least one environment.

^a^ In the case of the IO population, PH was measured in 2011 and 2012

^b^ *T* ‘TRAP#1/BOW//Taigu derivative’, *I* ‘Ivan/Soru#2’, *O* ‘Ocoroni F86’.

## Discussion

FHB index after field spray inoculation was generally considered as for a combination of Type I and Type II resistance; but in our study it appeared that mainly the former took place considering the significantly high correlation of FHB with PH and AE (Fig 1) that did not happen in point inoculated experiments for Type II resistance [11]. Therefore, we considered that the results obtained in this study were based mainly on Type I resistance.

Dwarfing genes *Rht-B1* and *Rht-D1* and the vernalisation gene *Vrn-A1* were segregating in both populations used in this study, resulting in that most of the phenotypic variation for FHB parameters was explained by these three loci, whereas other QTL only explained a small fraction of the variation (Table 3). The latter category comprised QTL with phenotypic effects below 10%, which were likely known QTL based on their locations [7].

The association between the two dwarfing genes and FHB susceptibility has been reported in many studies, and three possible mechanisms including disease escape, pleiotropy and tight linkage have been proposed; but a conclusion has not been drawn as to which mechanism was actually taking place. Intuitionally, this could be ascribed to PH per se or escape since tall plants were farther from soil surface where the inoculum was present (in the case of natural infection or spawn inoculation where FHB infected grain kernels were scattered in the field as inoculum) and ventilation was reduced that lead to high humidity favourable to FHB development [28, 49]. This mechanism must have contributed to the association in this study since the correlation remained significant in the sub-populations with homozygous *Rht-B1* and *Rht-D1* alleles (data not shown); despite the utilization of spray inoculation in this study, huge quantity of *Fusarium* inoculum was present on soil surface due to the adoption of wheat/maize rotation and conservation agricultural practices, supporting the escape mechanism. With the accumulation of molecular evidences in the last decade, more researchers took pleiotropy as the main mechanism for this association. Being DELLA protein producers, *Rht-B1b* and *Rht-D1b* have shown association with reduced resistance to biotrophic diseases including Type I FHB resistance (although FHB is regarded as a necrotrophic disease, it behaves more like a biotrophic disease at the early stages when Type I resistance takes place) but increased resistance to necrotrophic diseases like Type II FHB resistance [27]. Another evidence for the genetic effects of dwarfing genes instead of disease escape was that in sub-populations with homozygous *Rht* alleles, the correlation between PH and FHB disappeared or was significantly reduced [17, 21, 50], which was obviously not the case of the current study. However, this does not necessarily mean that the pleiotropic effects had no impacts on FHB in our study; it could function through controlling AE, which will be further explained below. Due to the limitation of map resolution, currently it is very difficult to separate pleiotropy and tight linkage; nevertheless clues supporting the latter have been reported. In the Soissons x Orvantis mapping population, Srinivasachary et al. [23] found that the peaks of FHB QTL were constantly located in a short distance away from the *Rht-D1* locus. Similarly in our previous research, a QTL for FHB in close linkage with *Rht-D1* appeared when PH was used as covariate [39]. So it appears that all the three mechanisms exist, but they are not necessarily simultaneously present in a single wheat line and their different combinations are expected.

The importance of AE in FHB resistance has long been recognized [29, 32], but genetic studies on AE were performed only in the last few years [8, 11, 38, 39]. In all these studies, the accumulated phenotypic variation explained by identified QTL for AE rarely exceeded 50% (usually around 30%), in accordance with the current study (Table 4), demonstrating a typical quantitative inheritance of AE. In the aforementioned four studies, totally 18 AE QTL have been identified, but only the QTL on 7AL found by Skinnes et al. [8] and Lu et al. [11] may be the same, and the one found on 4AL in He et al. [39] could be the same as reported by Buerstmayr and Buerstmayr [38]. In the current study, six more AE QTL were found (Table 4), and not unexpectedly only the one on 2DS might be the same as found in our previous study [39], whereas others were all from new chromosome regions. Similar to previous results, four out of the six AE QTL were associated with FHB resistance (Tables 3 and 4), supporting the phenotypic association of the two traits.

The two dwarfing genes showed consistent effects on reducing AE in both populations across environments, collectively contributing about 20% of AE variation. The association may have its physiological basis. In Arabidopsis, the elongation of anther filament is stimulated by GA and repressed by DELLA proteins which are orthologous to wheat *Rht-1* gene products [51]. Thus it is reasonable to speculate that the GA insensitive mutants *Rht-B1b* and *Rht-D1b* in wheat have similar function in repressing anther elongation through over expression of DELLA proteins, resulting in the phenotype of anther retention or low AE. This finding partly explained the pleiotropic effects of *Rht-B1b* and *Rht-D1b* on Type I FHB susceptibility, i.e. the two dwarfing genes lead to low AE, which in turn caused increased Type I FHB susceptibility. The results have also implications for hybrid wheat breeding, in which the selection of male parent, the pollen provider, is very important. A good male parent is expected to have high AE and high pollen production, and these two traits were reported to be significantly positively associated with *r* = 0.82 by Joppa et al. [52] and was later validated by Johnson and Patterson [53] and Atashi-Rang and Lucken [54]. Thus the utilisation of wild type *Rht* alleles *Rht-B1a* and *Rht-D1a* will improve both traits. Still more, the tall stature of such lines is favourable for efficient pollination, since it was suggested that the male parent be taller than the female parent in hybrid seed production [55].

## Supporting Information

S1 Table Linkage map information for the ‘TRAP#1/BOW//Taigu derivative’ x ‘Ocoroni F86’ population. (TXT)

S2 Table Linkage map information for the ‘Ivan/Soru#2’ x ‘Ocoroni F86’ population. (TXT)
